# Canopy light cues affect emission of constitutive and methyl jasmonate-induced volatile organic compounds in *Arabidopsis thaliana*

**DOI:** 10.1111/nph.12407

**Published:** 2013-07-12

**Authors:** Wouter Kegge, Berhane T Weldegergis, Roxina Soler, Marleen Vergeer-Van Eijk, Marcel Dicke, Laurentius A C J Voesenek, Ronald Pierik

**Affiliations:** 1 Plant Ecophysiology, Institute of Environmental Biology, Utrecht University3584 CH, Utrecht, the Netherlands; 2 Laboratory of Entomology, Wageningen UniversityPO Box 8031, 6700 EH, Wageningen, the Netherlands

**Keywords:** *Arabidopsis thaliana*, herbivory, light quality, phytochrome, shade avoidance, volatile organic compounds (VOCs)

## Abstract

- The effects of plant competition for light on the emission of plant volatile organic compounds (VOCs) were studied by investigating how different light qualities that occur in dense vegetation affect the emission of constitutive and methyl-jasmonate-induced VOCs.
- *Arabidopsis thaliana* Columbia (Col-0) plants and *Pieris brassicae* caterpillars were used as a biological system to study the effects of light quality manipulations on VOC emissions and attraction of herbivores. VOCs were analysed using gas chromatography–mass spectrometry and the effects of light quality, notably the red : far red light ratio (R : FR), on expression of genes associated with VOC production were studied using reverse transcriptase–quantitative PCR.
- The emissions of both constitutive and methyl-jasmonate-induced green leaf volatiles and terpenoids were partially suppressed under low R : FR and severe shading conditions. Accordingly, the VOC-based preference of neonates of the specialist lepidopteran herbivore *P. brassicae* was significantly affected by the R : FR ratio.
- We conclude that VOC-mediated interactions among plants and between plants and organisms at higher trophic levels probably depend on light alterations caused by nearby vegetation. Studies on plant–plant and plant–insect interactions through VOCs should take into account the light quality within dense stands when extrapolating to natural and agricultural field conditions.

## Introduction

Plants acclimate to their dynamic environment, and this requires both the ability to sense the environment and a signalling network via which to respond to these environmental changes. When plants grow in dense stands, as is the case in most agricultural and productive natural fields, they will have to deal with the close proximity of neighbouring plants that compete for resources. Aboveground, plants can sense neighbours through alterations in the light intensity and quality. Plants typically absorb red and blue light for photosynthesis, whereas far-red is reflected and transmitted through neighbouring leaves. Changes in the ratio between red and far-red light (the R : FR ratio) are the first light cues associated with imminent shading, and plants can detect these changes using their phytochrome photoreceptors (reviewed by Franklin, 2008). The light spectrum of horizontally reflected light is already FR-enriched before true shading occurs, as a result of FR reflection by neighbouring plants, which is sensed as an early neighbour detection signal (Ballaré *et al.*, 1990). At the physiological level, plants respond to a lowering of the R : FR ratio with a broad range of responses, including enhanced stem and petiole elongation, apical dominance and early flowering, which constitute the so-called shade avoidance syndrome (reviewed in Ballaré, 1999; Franklin, 2008; Martinez-Garcia *et al.*, 2010; Keuskamp *et al.*, 2011). These responses help plants to consolidate a favourable position in dense stands to support light capture and sustain their growth. In addition to light signals, volatile organic compounds (VOCs) have also been hypothesized to be involved in plant neighbour detection during competition (Kegge &Pierik, 2010), but this has been experimentally shown only for the volatile plant hormone ethylene (Pierik *et al.*, 2003, 2004). The production of ethylene is generally stimulated by low R : FR conditions (Finlayson *et al.*, 1999; Pierik *et al.*, 2004, 2009; Foo *et al.*, 2006; Kurepin *et al.*, 2007). It remains to be demonstrated whether and how emissions of VOCs other than ethylene are affected by the light conditions in stands with high plant densities.

Competing neighbours are not the only organisms that can threaten a plant's potential for growth and reproduction in dense stands. Particularly at high densities, plants are at risk of being attacked by herbivorous insects, and plants have evolved various mechanisms to fend off herbivores. Studies on simultaneous exposure to competing neighbours and herbivore attack have led to the hypothesis of a trade-off between shade-induced growth responses and plant defences (Cipollini, 2004). This conflict between growth and defence has also been coined ‘the dilemma of plants’ (Herms &Mattson, 1992; Ballaré, 2009). Indeed, mechanistic studies focussing on shade avoidance and defence showed that low R : FR conditions lead to a severe suppression of inducible plant defences in *Nicotiana longiflora* and *Arabidopsis thaliana* against herbivores (Izaguirre *et al.*, 2006; Moreno *et al.*, 2009), as well as against pathogens (Cerrudo *et al.*, 2012; reviewed in Ballaré *et al.*, 2012). Defences against herbivore attack are induced upon insect feeding through increased production of jasmonic acid (JA; Baldwin *et al.*, 1997; Dicke *et al.*, 1999). Accordingly, treatment of plants with exogenous JA induces various defence responses (Pieterse *et al.*, 2009), and the JA receptor mutant *coi1-1* (coronatin insensitive protein1-1) displays an enhanced susceptibility to herbivorous insects (Reymond *et al.*, 2004; Bodenhausen &Reymond, 2007; Van Oosten *et al.*, 2008). JA is not only involved in direct defence against herbivores, but also plays a significant role in the production of herbivore-induced plant volatiles (HIPVs) in response to herbivore attack (Dicke *et al.*, 1999; Snoeren *et al.*, 2009). Exogenous application of JA induces the emission of different volatile classes, such as green leaf volatiles (GLVs), phenylpropanoids/benzenoids and mono-, di- and sesquiterpenes (Snoeren *et al.*, 2009). Moreover, methyl jasmonate (MeJA), the methylated and volatile form of JA, is effective in inducing direct defences in plant leaves (Farmer &Ryan, 1990; Miksch &Boland, 1996) and inducing volatile emissions (Chen *et al.*, 2003; Faldt *et al.*, 2003; Bruce *et al.*, 2008; Herde *et al.*, 2008).

The emission of HIPVs is considered to serve as an indirect defence mechanism because HIPVs can attract natural enemies of herbivores (Vet &Dicke, 1992; Dicke &Baldwin, 2010). However, herbivores and plants can also exploit HIPVs: herbivores may avoid oviposition on plants that already contain eggs or feeding herbivores (Dicke, 2000), while specialist herbivores may be attracted by HIPVs (Bolter *et al.*, 1997) and plants grown near damaged neighbours may become more resistant to herbivory, as was shown for tobacco (*Nicotiana tabacum*) grown near damaged sagebrush (*Artemisia tridentata*) (Karban *et al.*, 2003). JA-deficient mutants display a reduced induction of many volatiles upon herbivore attack, especially terpenoids and GLVs (Thaler *et al.*, 2002; Snoeren *et al.*, 2009). Accordingly, JA-deficient tomato (*Lycopersicon esculentum*) mutants receive more oviposition from herbivorous insects than do wild-type plants (Sanchez-Hernandez *et al.*, 2006). These findings indicate that JA controls VOC-mediated interactions between plants and herbivores.

Because interplant distances in dense stands are small, the likelihood of VOCs emitted by a plant reaching physiologically meaningful concentrations at its neighbours, and this would favour eavesdropping on neighbouring plants to detect, for example, upcoming herbivore attack. However, the emission of many VOCs relies on JA signalling, and the low R : FR conditions in a dense stand reduce JA-mediated defences. We therefore investigated in *Arabidopsis thaliana* whether (1) low R : FR, green shade (mimicking light conditions at early and late stages of canopy development, respectively) and dense vegetation affect constitutive VOC emissions; (2) low R : FR affects MeJA-induced VOC emissions; and (3) low R : FR affects localization of the food plant by naïve newly hatched caterpillars (neonates) of *Pieris brassicae*, a specialist herbivore of brassicaceous plants such as *A. thaliana*. We demonstrate that the emission of several constitutively emitted VOCs is reduced under low R : FR conditions, green shade and dense canopy conditions. Furthermore, we show that the emission of MeJA-induced VOCs is also reduced when plants are grown in low R : FR and that plant preference of *P. brassicae* based on VOCs is impeded. We conclude that light quality can have a strong impact on the emission of plant VOCs and their role in biotic interactions.

## Materials and Methods

### Plant growth conditions and insect rearing

**Fig 1:**
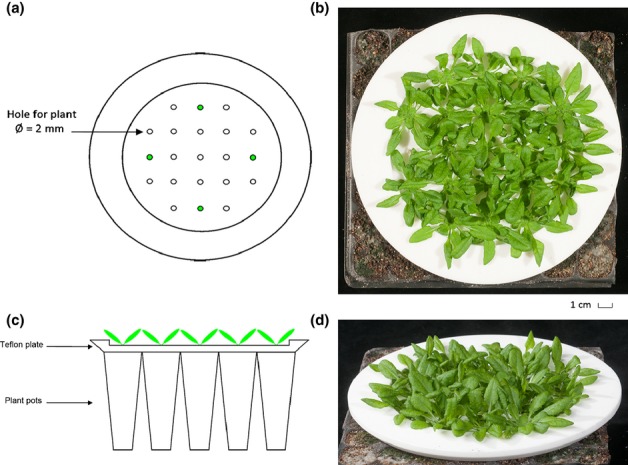
Graphic representation of the Teflon plate used to hold plants for volatile collection. (a) Top view of the plate (inner diameter 131 mm; outer diameter 183 mm); green spots indicate the holes that were used for individual plants. (b) Top view of the plant canopy. (c) Side view of the plate (plate thickness 3 mm) placed on top of the plant pots, with plants growing through it. (d) Side view of the plant canopy.

*Arabidopsis thaliana* (L.) Heynh accession Columbia (Col-0) plants were grown in a 1 : 2 potting soil : perlite mixture enriched with 0.14 mg of MgOCaO (17%; Vitasol BV, Stolwijk, the Netherlands) and 0.14 mg of slow-release fertilizer (Osmocote Plus Mini; Scotts International, Heerlen, the Netherlands) per pot (70 ml; Millenaar *et al.*, 2009). After sowing, seeds were stratified for 3 d (in the dark at 4°C) and subsequently placed in a growth room (with short days (9 h light, 15 h dark), 150 μmol m^−2^ s^−1^ photosynthetically active radiation (PAR; Philips HPI-T Plus, Philips, Eindhoven, the Netherlands), a temperature of 20°C and 70% relative humidity) and kept underneath a glass plate to prevent seedling dehydration. After 3 d, the glass plate was removed and pots were placed on automatically watered mats. Seedlings were transferred to individual pots (containing 70 ml of soil mixture) after 9–11 d. For volatile collection, plants were transferred to individual pots (containing 19 ml of soil : perlite mixture) that were covered with a Teflon plate with 1-mm holes to fit the hypocotyl through (Fig.1) and to separate the shoot compartment from the pot and soil. In high plant density experiments, plants were also grown in these 10-ml pots and these were positioned in square grids to reach a density of 2066 plants m^−2^.

The herbivorous large cabbage white *Pieris brassicae* L. was reared on Brussels sprouts (*Brassica oleracea* L. var. *gemmifera*, cv Cyrus) plants in a growth chamber (16 h : 8 h, light : dark, 20°C and 70% relative humidity). Eggs were laid on *B. oleracea* leaves, but removed from leaves *c*. 18–24 h before hatching.

### Experimental approaches

Light treatments were created in the following ways. Plants were exposed to a low red (λ = 660 nm) : far-red (λ = 730 nm) ratio (R : FR) in a growth chamber compartment with supplemental FR LEDs (730 nm; Philips Green Power) in a control white-light background of 150 μmol m^−2^ s^−1^ PAR. This reduced the R : FR ratio from 2.2 (control white light) to 0.2 (measured with the Skye SKR 100 R : FR detector; Skye Instruments, Llandrindod Wells, UK). Control plants were placed in a similar compartment with the same light intensity and the standard R : FR ratio of 2.2 produced by the background white lamps. Green shade was achieved using a green, light-absorbing filter (Lee 122 Fern Green; Lee, Hampshire, UK), which reduced PAR to 90 μmol m^−2^ s^−1^ and R : FR to 0.25.

MeJA was used to induce herbivory-associated defence in a homogenous and standardized manner (Dicke *et al.*, 1999; Wu *et al.*, 2008; Moreno *et al.*, 2009). The effects of MeJA were studied on plants that were grown in control light and in plants that had been exposed to low R : FR conditions for 6 d. MeJA (pure quality; Van Meeuwen Chemicals BV, Weesp, the Netherlands) was sprayed onto the shoots at a concentration of 100** **μM MeJA, which is a standard concentration and mode of application often used to induce JA pathways in *A. thaliana* (Leon-Reyes *et al.*, 2009; De Wit *et al.*, 2013), that was dissolved in 0.02% Silwet to facilitate an equal spread of the solution over the leaves. Mock-treated plants were sprayed with 0.02% Silwet (http://www.momentive.com). Per plant, *c*. 1 ml of solution was sprayed. Plants were placed in the fume hood to dry for 1 h and then moved back into the growth chamber. MeJA was sprayed onto plants 24 h before volatile collection, harvesting for gene expression or choice assays with caterpillars.

### Dynamic headspace collection of plant volatiles

Plants were grown in the Teflon plate-covered pots for 33–35 d at a low density (394 plants m^−2^; no plant–plant interactions through touch or light signals) or a high density (2066 plants m^−2^; strong plant–plant interactions) of plants per plate. Plates had a diameter of 131 mm, with holes organized in square grids, as shown in Fig.1a (inter-hole distance is 25 mm). The holes had a diameter of 2 mm, and the hole depth was 3 mm. Volatile samples were taken from aboveground plant parts; the Teflon plate prevented interference from belowground VOCs (Fig.1). Any unused holes in the Teflon plate were sealed with a few drops of 2% agarose gel. Air entering the cuvette (volume: 1.8 l) was first cleaned through an activated charcoal filter (4/8 mesh; Sigma Aldrich NL, Zwijndrecht, the Netherlands) and a Tenax TA cartridge (Markes, Llantrisant, UK). After 30 min of flushing, plant headspace collection occurred for 4 h at a flow rate of 4 l h^−1^ by trapping on a Tenax TA cartridge containing 200 mg of Tenax-TA (20/35 mesh; Grace-Alltech, Deerfield, MI, USA). Volatile collection was performed at room temperature (21 ± 1°C).

### Ethylene production

Ethylene production was measured on freshly harvested shoot tissue (*c*. 1.0–1.2 g fresh weight) after 20 min of headspace accumulation in a syringe, as previously described (Millenaar *et al.*, 2005), using a GC955 gas chromatograph with photo-ionization detector and a 160-cm Haye Sep R column, filled with Haye Sep 80/100 mesh (Synspec, Groningen, the Netherlands).

### Analysis of plant volatiles

A Thermo Trace GC Ultra (Thermo Fisher Scientific, Waltham, MA, USA) coupled with a Thermo Trace DSQ (Thermo Fisher Scientific) quadrupole mass spectrometer (MS) was used for separation and identification of plant volatiles. Before releasing volatiles, the Tenax TA cartridges were dry-purged under a flow of nitrogen at 20 ml min^−1^ for 10 min at ambient temperature in order to remove moisture. Compounds from the compressed air used as a carrier for volatiles during their collection and those from empty plant growth cuvets were collected and, together with volatiles from clean Tenax TA adsorbents and the analytical system itself, these were subtracted from the final data as a corrective measure. The collected volatiles were thermally released from the Tenax TA in a thermal desorption unit (Ultra 50 : 50; Markes) at 250°C for 10 min under a helium flow of 20 ml min^−1^ while re-collecting the volatiles in a thermally cooled universal solvent trap (Unity; Markes) at 10°C. Once the desorption process had been completed, the cold trap was heated ballistically from 40 to 280°C and was held for 10 min while the volatiles were transferred to a ZB-5MSi analytical column (30 m × 0.25 mm I.D. × 1.00 μm F.T.; Phenomenex, Torrance, CA, USA), in a splitless mode for separation. The analytical column was set at an initial temperature of 40°C and immediately raised at 5°C min^−1^ to 280°C and was held for 4 min under a column flow of 1 ml min^−1^ in a constant flow mode. The DSQ MS was operated in a scan mode with a mass range of 35–350 amu at 5.38 scans s^−1^ and ionization was performed in EI mode at 70 eV. The MS transfer line and ion source were set at 275 and 250°C, respectively. Identification of compounds was based on comparison of mass spectra with those in the NIST 2005 and Wageningen University Mass Spectral Database of Natural Products. Experimentally calculated linear retention indices (LRIs) were used as an additional criterion for confirming the identity of compounds. Relative quantification (peak areas of individual compounds) was carried out using a single (target) ion in selected ion monitoring (SIM) mode. These individual peak areas of each compound were corrected for plant fresh weight and further used for characterization of the different treatment groups using statistical analysis.

### Choice assays

The innate preference of neonate *P. brassicae* caterpillars was evaluated in two-choice experiments (Soler *et al.*, 2012). Eggs of *P. brassicae*, laid on Brussels sprouts plants (*B. oleracea*), were removed *c*. 18–24 h before hatching. This was done to obtain naïve neonates that had not fed on plant material before the experiment. Newly emerged neonates were individually placed on carton platforms (1 cm^2^), bridging control and treatment plants. Neonate caterpillars were released individually in the middle of the platform facing north, with the two choice plants located to the east and west (with control and treatment alternating between east and west between replicates). Care was taken that lighting conditions were uniform and that the light was from above, such that this would not directionally influence caterpillar choice. Caterpillars were then observed continuously until a choice was made (maximally 15 min). A choice was recorded when the larva climbed onto a plant. To avoid the induction of plant defences and thereby a possible influence on the choice of subsequently released conspecifics, larvae were removed from the leaves before they started feeding. A minimum of eight plant pairs and 18 larvae per pair were tested per combination of treatments. The following plant treatments were compared on 30–33*-*d-old plants: control versus low R : FR; control versus MeJA; control versus low R : FR + MeJA; low R : FR versus low R : FR + MeJA; and MeJA versus low R : FR + MeJA. Plants were placed in low R : FR conditions 6 d before the choice assays, while *c*. 1 ml of 100 μM MeJA was sprayed per plant 24 h before choice assays.

### RNA isolation and reverse transcriptase–quantitative PCR (RT-qPCR)

Leaf laminas of three fully grown leaves were snap-frozen in liquid nitrogen and subsequently stored at −80°C. Total RNA was extracted using the Qiagen RNeasy Plant Mini Kit with on-column DNase treatment. cDNA was synthesized using 100 units of SuperScript III reverse transcriptase (Invitrogen) with random hexamers at 50°C in a reaction volume of 20 μl. RT-qPCR was performed in a BioRad MyIQ single-colour real-time PCR detection system using SYBR Green Supermix (BioRad, Hercules, CA, USA). The Primer3Plus software (Untergasser *et al.*, 2007) was used to develop gene-specific primers and the 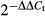 method was used to calculate relative gene expression (Livak &Schmittgen, 2001) with *Ubiquitin 5* (*UBQ5*) and *Tubulin 6* (*TUB6*) as internal standards. Primer sequences are shown in Supporting Information Table S1. Primers were tested for gene specificity by performing melt curve analysis on a BioRad MyIQ single-colour RT-qPCR detection system using SYBR Green Supermix (BioRad). Expression of the following genes that are associated with VOC biosynthesis was measured: *VEGETATIVE STORAGE PROTEIN 2* (*VSP2*), which is known to code for a protein that slows herbivore feeding (Liu *et al.*, 2005); *TERPENE SYNTHASE 4* (*TPS4*), which is involved in the biosynthesis of geranyl linalool, a precursor of (E,E)-4,8,12-trimethyltrideca-1,3,7,11-tetraene (TMTT; Herde *et al.*, 2008); *TPS3*, another terpene synthase, which has been shown to be involved in the synthesis of β-ocimene and β-myrcene (Faldt *et al.*, 2003); *CYP72A13* (*CYTOCHROME P450 72A13*), which is postulated to be involved in the conversion of geranyl linalool to TMTT (Bruce *et al.*, 2008); and *BSMT1* (S-Adenosylmethionine-Dependent Methyltransferase), which is involved in the methylation of salicylic acid (SA) and benzoic acid (Chen *et al.*, 2003).

### Statistical analysis

Headspace compositions were statistically analysed in a multivariate analysis using simca p+ ver. 12.0.1.0 (Umetrics, Umeå, Sweden). The quantitative results for the volatile blends in the different treatments were log-transformed, mean-centred and scaled to unit variance before being analysed using a multivariate approach called projection to latent structures–discriminant analysis (PLS-DA). PLS-DA with a classical PLS regression takes advantage of class information in maximizing the separation between groups of observations, where the dependent variable *y* is categorical and represents sample class membership (Szymańska *et al.*, 2012). In PLS-DA, cross-validation is employed in order to determine the number of significant PLS components (Eriksson *et al.*, 2006). Results of PLS-DA analysis can be displayed as a score plot, where pattern recognition of sample groups can be visualized. The score plot can be complemented by a loading plot, which can show which *x*-variables, in this case volatile compounds, are playing a role in making the group separation of samples in the score plot possible.

Data on peak area units were statistically analysed using a one- or two-way ANOVA, followed by a Bonferroni post hoc test to allow comparisons between different treatments. For herbivore choice experiments, data were analysed with a binominal test. These comparisons were made using IBM spss statistics 20 software (IBM, Amsterdam, the Netherlands).

## Results

### Volatile emission by *Arabidopsis thaliana* decreases with increasing shade

To determine how proximate neighbours and their effects on light quality and quantity affected the emission of VOCs, individually grown plants were placed in three different light conditions: control light, low R : FR light (mimicking close, but not yet shading neighbours), and green shade (mimicking shade imposed by neighbours). As a fourth treatment, plants were grown in a dense stand. Under these four conditions, VOCs were collected for 4 h and analysed by gas chromatography–mass spectrometry (GC-MS). Volatiles were quantified relatively by calculating the average peak area per compound per treatment. When plants were exposed to light conditions that occur at different stages of canopy development (low R : FR early on and green shade at later stages) or to high plant density, overall volatile emission after 4 h of sampling decreased.

**Fig 2:**
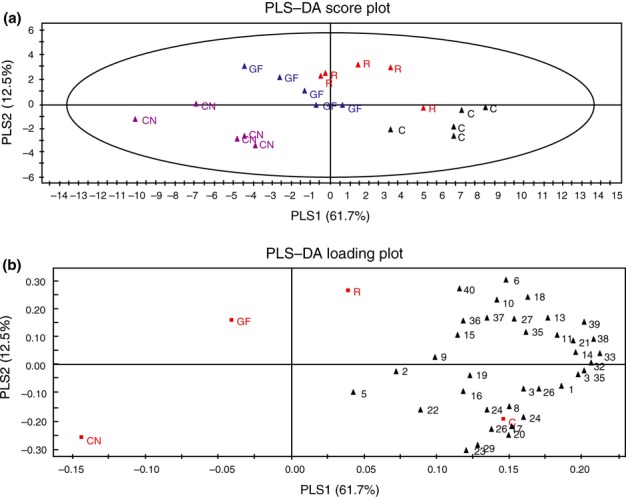
Projection to latent structures–discriminant analysis (PLS-DA) of volatile emissions produced by *Arabidopsis thaliana* plants in four different treatments: control plants (C), plants exposed to low red (R) : far-red (FR) (R), plants under a green filter (GF) and plants that were growing in an actual canopy (CN). A score plot (a) and loading plot (b) of the first two principal components, with the explained variance in brackets, are shown. The ellipse defines the Hotelling's T2 confidence region (95%). Numbers in the loading plot (b) correspond to numbers representing individual volatile compounds as presented in Table 1.

In order to distinguish if treatments cluster as groups, data were analysed with a PLS-DA (Fig.2). The first two principal components explained 61.7% and 12.5% of the variance, respectively. The headspace composition of canopy-grown plants was most closely related to the headspace of plants exposed to green shade. The headspace composition of control plants was most closely related to the headspace of plants exposed to low R : FR conditions.

**Fig 3:**
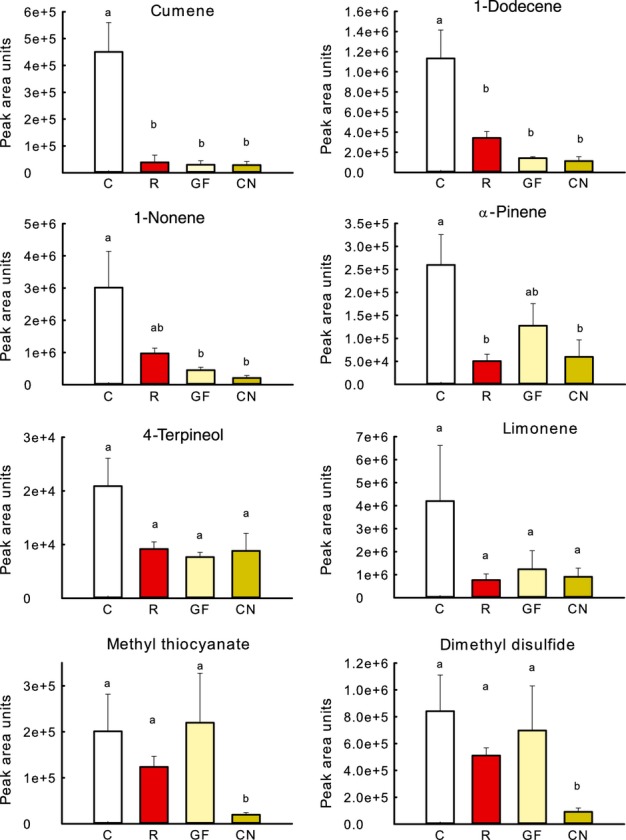
Emission of different volatile organic compounds (VOCs) by undamaged *Arabidopsis thaliana* plants, in terms of peak area units per gram plant dry weight detected in gas chromatography–mass spectrometry (GC-MS) analysis. Bars represent mean ± SE (*n *=* *5). Volatiles were detected in the headspace of undamaged *A. thaliana* plants collected for 4 h. Volatiles were collected from control plants (C), plants exposed to low red (R) : far-red (FR) (R), plants under a green filter (GF) and plants that were growing in an actual canopy (CN). Significant differences are represented by different letters (*P *<* *0.05; one-way ANOVA; Bonferroni post hoc).

**Fig 4:**
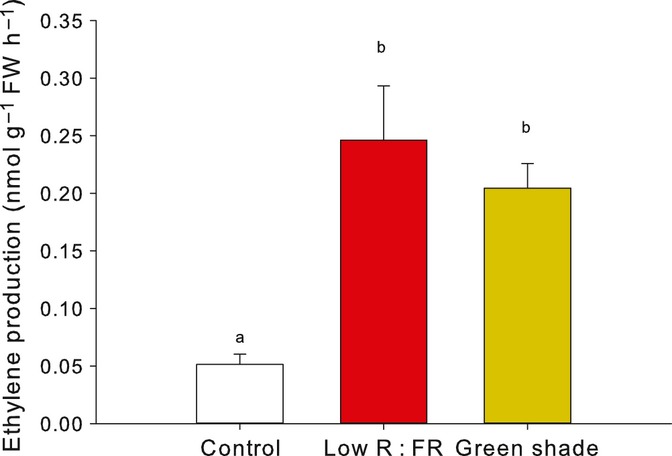
Ethylene emission of control, low red (R) : far-red (FR) and green filter treated *Arabidopsis thaliana* plants after 4 h of treatment. Bars represent mean ± SE (*n *=* *7). Different letters indicate significant differences (one-way ANOVA; Bonferroni post hoc).

In Table 1, per volatile compound the different treatments are ranked based on the average peak area units (representing the relative amount of emission by plants) in the respective treatments. In columns 3–6, the four different treatments are arranged from highest emission (left, column 3) to lowest emission (right, column 6). Considering the peak area units per compound, the peak area for the control was in 12 out of 40 cases significantly higher than for all the other three treatments, whereas the emission of these 12 compounds was not different among the three treatments. In Fig.3, results for cumene and 1-dodecene illustrate the above-described pattern: both volatiles had higher emission rates in the control treatment than in any of the other treatments, while no difference in emission rate between the different shade treatments was observed. No compounds with significantly higher emission in any of the light/density treatments than in control plants were detected (Table 1). For 2-pentanone, 1-heptene, pentadecanal and 1-nonene, the peak area for control plants was higher than for plants exposed to green shade or for canopy plants, while no difference in emission was found between control and low R : FR-treated plants or between low R : FR and green shade/canopy plants (Table 1); this pattern is illustrated for 1-nonene in Fig.3. Emission of α-pinene was reduced in the low R : FR and canopy treatments compared with control plants, while no difference was detected between control and green-shade-treated plants (Fig.3). In 23 out of 40 cases, average peak areas ranked as control > low R : FR > green shade > canopy (Table 1), and this frequency of occurrences is significantly higher than expected (*P *<* *0.001; χ^2^ test). However, in nine of these 23 cases no significant difference in peak area was observed between the treatments, as illustrated for 4-terpineol and limonene in Fig.3. Consistent with the observation that in canopies the emission of ethylene is increased (De Wit *et al.*, 2012), the ethylene emission increased under low R : FR conditions and under green shade (Fig.4).

**Table 1:** Volatile compounds detected in the headspace of *Arabidopsis thaliana* plants listed according to their elution order

No.	Compound	Ranking			
1	3-Methyl-2-butanone	Control^a^	Low R^b^	Green filter^b^	Canopy^b^
2	1-Penten-3-ol	Control	Green filter	Canopy	Low R
3	2-Pentanone	Control^a^	Low R^ab^	Green filter^b^	Canopy^b^
4	1-Heptene	Control^a^	Low R^ab^	Green filter^b^	Canopy^b^
5	3-Pentanone	Control	Canopy	Low R	Green filter
6	Methyl thiocyanate	Green filter	Control	Low R	Canopy
7	2-Methyl-butanenitrile	Control^a^	Low R^ab^	Green filter^ab^	Canopy^b^
8	Methyl-cyclohexane	Control^a^	Green filter^b^	Low R^b^	Canopy^b^
9	3-Methylbutanenitrile	Control	Low R	Green filter	Canopy
10	Dimethyl disulfide	Control	Green filter	Low R	Canopy
11	1-Octene	Control	Low R	Green filter	Canopy
12	(R)-3-Methylcyclopentanone	Control^a^	Low R^b^	Green filter^b^	Canopy^b^
13	Cyclohexanol	Control	Low R	Green filter	Canopy
14	1-Nonene	Control^a^	Low R^ab^	Green filter^b^	Canopy^b^
15	Cyclohexanone	Control	Low R	Green filter	Canopy
16	Anisole	Control	Green filter	Low R	Canopy
17	Cumene	Control^a^	Low R^b^	Green filter^b^	Canopy^b^
18	2-Cyclohexen-1-one	Control	Low R	Green filter	Canopy
19	α-Pinene	Control^a^	Green filter^ab^	Canopy^b^	Low R^b^
20	Propylbenzene	Control^a^	Canopy^b^	Green filter^b^	Low R^b^
21	6-Methyl-5-hepten-2-one	Control^a^	Low R^ab^	Green filter^ab^	Canopy^b^
22	Limonene	Control	Green filter	Canopy	Low R
23	m-Cymene	Control^a^	Canopy^b^	Green filter^b^	Low R^b^
24	2-Isopropenyl-5-methylhex-4-enal	Control	Low R	Green filter	Canopy
25	1-Undecene	Control^a^	Low R^b^	Green filter^b^	Canopy^b^
26	2-Butanoylfuran	Control^a^	Low R^ab^	Canopy^ab^	Green filter^b^
27	3-Acetyl-2,5-dimethylfuran	Control	Low R	Green filter	Canopy
28	Pulegone	Control^a^	Green filter^b^	Canopy^b^	Low R^b^
29	Isodurene	Control^a^	Canopy^b^	Green filter^b^	Low R^b^
30	4-Terpineol	Control	Low R	Canopy	Green filter
31	1-Dodecene	Control^a^	Low R^b^	Green filter^b^	Canopy^b^
32	1-Tridecene	Control^a^	Low R^b^	Green filter^b^	Canopy^b^
33	1-Tetradecene	Control^a^	Low R^b^	Green filter^b^	Canopy^b^
34	Longifolene	Control^a^	Canopy^ab^	Low R^b^	Green filter^b^
35	Neoclovene	Control	Low R	Green filter	Canopy
36	(*E,E*)-α-Farnesene	Low R	Control	Green filter	Canopy
37	(*E,E*)-TMTT	Control	Green filter	Low R	Canopy
38	1-Hexadecene	Control^a^	Low R^b^	Green filter^b^	Canopy^b^
39	Pentadecanal	Control^a^	Low R^ab^	Green filter^b^	Canopy^b^
40	(*E,E*)-TMMHT	Control	Low R	Green filter	Canopy

Volatile emission is ranked from high (left) to low (right) within each row, where significant differences (*P *<* *0.05; one-way ANOVA with Bonferroni post hoc test) are indicated by different letters. Volatiles were collected from control plants (Control), plants exposed to low red : far-red (Low R), plants under a green filter (Green filter) and plants that were growing in an actual canopy (Canopy). (*E,E*)-TMTT, (*E,E*)-4,8,12-trimethyltrideca-1,3,7,11-tetraene; (*E,E*)-TMMHT, (*E,E*)-7,11,15-trimethyl-3-methylene-hexadeca-1,6,10,14-tetraene.

### MeJA-induced volatile emission is suppressed under low R : FR

**Fig 5:**
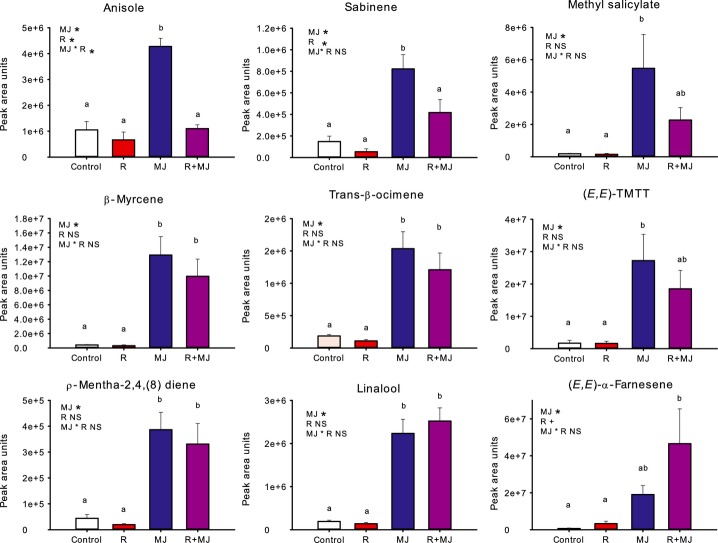
Peak area units of different volatile organic compounds (VOCs) emitted by *Arabidopsis thaliana*, corrected for plant dry weight. Volatiles were collected during 4 h from control plants (Control), plants exposed to low red (R) : far-red (FR) (R), plants sprayed with 100 μM methyl jasmonate (MJ) and plants exposed to low R : FR and treated with 100 μM methyl jasmonate (R+MJ). The text at the top left of each graph shows the main effects and interaction effect based on two-way ANOVA: * indicates a significant difference (*P *<* *0.05); + indicates a trend (0.10 > *P *>* *0.05); NS, not significant. Bars represent mean (*n *=* *5) ± SE. Significantly different classes are indicated by different letters (one-way ANOVA; Bonferroni post hoc).

**Fig 6:**
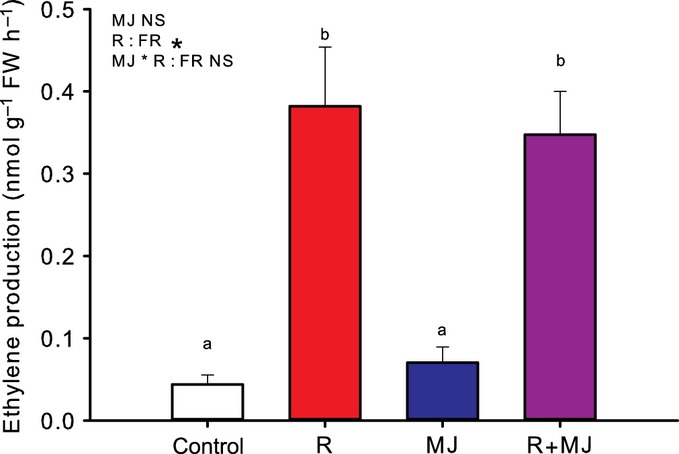
Low red (R) : far-red (FR)- and methyl jasmonate (MJ)-induced emission of ethylene from the shoot of *Arabidopsis thaliana* plants. Volatiles were collected from control plants (Control), plants exposed for 6 d to low R : FR (R), plants sprayed 24 h before ethylene measurements with 100 μM MJ (MJ) and plants exposed for 6 d to low R : FR and sprayed 24 h before ethylene measurements with 100 μM MJ (R+MJ). Data represent mean (*n *=* *5) ± SE. The text at the top left of each graph shows the outcome of two-way ANOVA testing: * indicates a significant difference (*P *<* *0.05); NS, not significant. Significantly different classes are indicated by different letters (one-way ANOVA; Bonferroni post hoc).

To test whether high density and canopy signals also affect MeJA-induced VOCs, VOC emission in plants that received a MeJA treatment, low R : FR treatment or MeJA + low R : FR double treatment was measured. In Table 2, per volatile compound the different treatments are ranked based on the average peak area units in the respective treatments. In columns 2–5, the four different treatments are arranged from highest emission (left, column 2) to lowest emission (right, column 5). For seven compounds, the emission in MeJA-treated plants under control light conditions was significantly higher than in MeJA-treated plants that were also exposed to low R : FR conditions. In control light, MeJA treatment significantly increased the emission of eight compounds (Table 2, Fig.5). MeJA did not induce the emission of four of these compounds (anisole, sabinene, methyl salicylate and TMTT) when plants had been exposed to low R : FR conditions. (E,E)-α-farnesene was the only compound significantly induced by MeJA under low R : FR that was not MeJA-induced in normal light conditions. The application of MeJA did not affect the emission of ethylene, irrespective of the light quality, whereas low R : FR did stimulate ethylene emissions (Fig.6).

**Table 2:** Volatile compounds detected in the headspace of *Arabidopsis thaliana* plants collected for 4 h, listed according to their elution order

Compound	Ranking			
2-Butenal	Low R + MeJA	MeJA	Control	Low R
1-Penten-3-ol	Control	MeJA	Low R + MeJA	Low R
1-Methoxy-2-propanone	Control	MeJA	Low R	Low R + MeJA
3-Pentanone	MeJA	Control	Low R + MeJA	Low R
Unknown	MeJA	Control	Low R	Low R + MeJA
2-Methyl-butanenitrile	MeJA^a^	Control^ab^	Low R + MeJA^b^	Low R^b^
Methyl-cyclohexane	MeJA^a^	Control^ab^	Low R^b^	Low R + MeJA^b^
Butyl acetate	MeJA^a^	Control^ab^	Low R^b^	Low R + MeJA^b^
1-Nonene	MeJA	Control	Low R	Low R + MeJA
(*E,E*)-2,4-Hexadienal	Low R + MeJA	Control	MeJA	Low R
Anisole	MeJA^a^	Low R + MeJA^b^	Control^b^	Low R^b^
1S-α-Pinene	MeJA^a^	Control^ab^	Low R + MeJA^ab^	Low R^b^
Sabinene	MeJA^a^	Low R + MeJA^b^	Control^b^	Low R^b^
β-Myrcene	MeJA^a^	Low R + MeJA^a^	Control^b^	Low R^b^
Unknown	MeJA	Control	Low R + MeJA	Low R
Limonene	MeJA	Control	Low R	Low R + MeJA
Phenylmethanol	Control	MeJA	Low R	Low R + MeJA
trans-β-Ocimene	MeJA^a^	Low R + MeJA^a^	Control^b^	Low R^b^
p-Mentha-2,4(8)-diene	MeJA^a^	Low R + MeJA^a^	Control^b^	Low R^b^
Linalool	Low R + MeJA^a^	MeJA^a^	Control^b^	Low R^b^
3-Acetyl-2,5-dimethylfuran	MeJA	Control	Low R	Low R + MeJA
(Z)-Tagetone	MeJA^a^	Control^ab^	Low R^b^	Low R + MeJA^b^
Alloocimene	MeJA	Control	Low R + MeJA	Low R
p-Methyl-acetophenone	MeJA	Control	Low R	Low R + MeJA
1-Dodecene	MeJA	Control	Low R	Low R + MeJA
Methyl salicylate	MeJA^a^	Low R + MeJA^ab^	Control^b^	Low R^b^
Indole	Low R + MeJA	MeJA	Control	Low R
2-Methylpropyl benzoate	MeJA^a^	Control^ab^	Low R^ab^	Low R + MeJA^b^
α-Copaene	MeJA	Control	Low R	Low R + MeJA
(*E,E*)-α-Farnesene	Low R + MeJA^a^	MeJA^ab^	Low Rb	Control^b^
Valencene	Control	Low R	MeJA	Low R + MeJA
γ-Cadinene	MeJA	Control	Low R + MeJA	Low R
(*E,E*)-TMTT	MeJA^a^	Low R + MeJA^ab^	Control^b^	Low R^b^
(*E,E*)-TMMHT	Low R + MeJA	MeJA	Control	Low R

Volatile emission is ranked from high (left) to low (right) within each row, where significant differences (*P *<* *0.05; one-way ANOVA with Bonferroni post hoc test) are indicated by different letters. Volatiles were collected from control plants (Control; white), plants exposed to low red : far-red (Low R; red), plants sprayed with 100 μM methyl jasmonate (MeJA; blue) and plants exposed to low red : far-red and treated with 100 μM MeJA (Low R + MeJA; purple). (*E,E*)-TMTT, (*E,E*)-4,8,12-trimethyltrideca-1,3,7,11-tetraene; (*E,E*)-TMMHT, (*E,E*)-7,11,15-trimethyl-3-methylene-hexadeca-1,6,10,14-tetraene.

**Fig 7:**
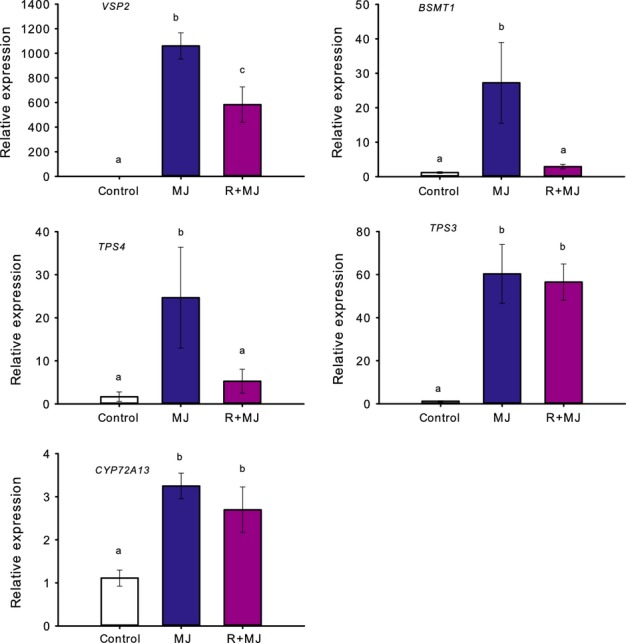
Relative expression of *VEGETATIVE STORAGE PROTEIN 2* (*VSP2*), S-adenosylmethionine-dependent methyltransferase (*BSMT1*), *TERPENE SYNTHASE 4* (*TPS4*), *TPS3* and *CYTOCHROME P450 72A13* (*CYP72A13*) in *Arabidopsis thaliana*. Control plants were grown under normal light conditions (red (R) : far-red (FR) 2.0), methyl jasmonate (MeJA) plants were sprayed with 100 μM MeJA 24 h before harvesting for gene expression (MJ), and double-treated plants were grown for 6 d at R : FR 0.2 and sprayed with 100 μM MeJA 24 h before harvesting for gene expression (R+MJ) (*n *=* *5). Data represent the mean of five independent biological replicates ± SE. Different letters indicate significant differences (one-way ANOVA; Bonferroni post hoc test).

To establish whether differences in MeJA-induced volatile emission between control and low R : FR-grown plants are associated with differences in transcriptional regulation, we tested several genes that are involved in the biosynthesis of HIPVs. In addition, we investigated the expression of the MeJA-responsive gene coding for vegetative storage protein 2 (VSP2) to confirm its previously described down-regulation under low R : FR (Moreno *et al.*, 2009) which is related to FR-mediated defence suppression. Indeed, *VSP2* expression was up-regulated after MeJA treatment and this up-regulation was suppressed by exposure to low R : FR conditions (Fig.7). Low R : FR had a negative effect on the MeJA-induced transcript levels of *TPS4* and *BSMT1* (Fig.7). MeJA-induced transcript levels of *CYP72A13* and *TPS3* were not reduced under low R : FR (Fig.7).

### Neonate *P. brassicae* preference for MeJA-treated plants is abandoned in low R : FR

**Fig 8:**
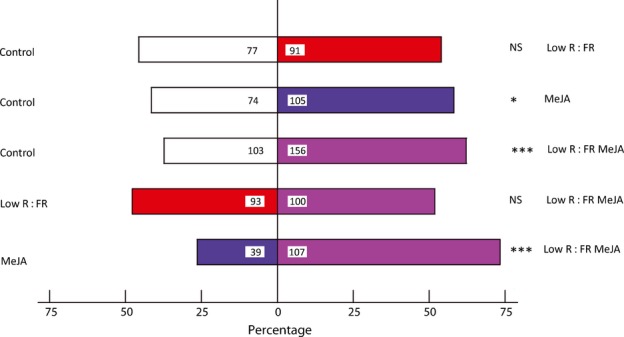
Preference of *Pieris brassicae* in two-choice tests. For each test, eight different *Arabidopsis thaliana* pairs were used. Plants grown at a red (R) : far-red (FR) ratio of 2.0 (Control), plants grown at an R : FR ratio of 0.2 (Low R : FR), plants sprayed with 100 μM methyl jasmonate (MeJA) and plants grown at an R : FR ratio of 0.2 and sprayed with 100 μM MeJA (Low R : FR MeJA) were tested. Bars represent the percentage of herbivores that preferred the treatment. Numbers in bars represent numbers of herbivores that made the corresponding choice. Data were analysed with a binominal test. Significant differences: *, *P *<* *0.05; ***, *P *<* *0.001; NS, no significant differences were found for the corresponding choice assays.

Because VOCs can serve as a cue for herbivores to locate their preferred host, we used a recently developed bioassay that uses orientation behaviour of *P. brassicae* caterpillars towards VOC emissions of plants (Soler *et al.*, 2012) and tested if this orientation behaviour is affected by R : FR light ratio, MeJA and their combination. When allowed to choose between control and low R : FR-grown plants, no preference of neonate *P. brassicae* caterpillars was observed (*P *=* *0.15; Fig.8). Low R : FR-grown plants sprayed with MeJA were preferred by 73% of the caterpillars over plants sprayed with MeJA and grown in normal light (*P *<* *0.001; Fig.8). Although 59% of the neonates of this specialist herbivore preferred plants treated with MeJA over mock-treated plants (*P *=* *0.012), this preference disappeared when plants were grown under low R : FR light; that is, low R : FR-grown plants treated with MeJA were not preferred over low R : FR-grown plants without MeJA treatment (*P *=* *0.33; Fig.8).

## Discussion

This study demonstrates that shade and shade-related plant cues from neighbouring plants reduce the emission of both constitutive and MeJA-induced volatile compounds in *A. thaliana*. Moreover, the preference of neonate *P. brassicae* caterpillars for MeJA-treated plants disappears when neonates have to choose between low R : FR-grown plants and low R : FR-grown plants that are sprayed with MeJA.

### Low R : FR light conditions modulate VOC emissions in dense stands

Because the root systems of plants at high density were kept separate, the effect of density on VOC emission was caused solely by aboveground factors. We therefore hypothesize that the reduction of VOC emission caused by growing at a high density was attributable to the combined effects of FR reflection by and transmission through leaves from neighbouring plants, and partial shading by neighbouring plants. Reduced volatile emission in green shade or dense canopy conditions is consistent with previous findings that VOC emissions are controlled by light intensity (Takabayashi *et al.*, 1994; Halitschke *et al.*, 2000; Maeda *et al.*, 2000; Gouinguene &Turlings, 2002). However, the reduction of VOC emissions in plants exposed to low R : FR without a reduction in light intensity implies that phytochrome signalling affects VOC emission. As phytochrome signalling controls JA responsiveness (Moreno *et al.*, 2009; Cerrudo *et al.*, 2012) and many herbivore-induced volatiles are produced through the JA pathway (Boland *et al.*, 1995; Dicke *et al.*, 1999; Van Poecke &Dicke, 2002; Halitschke *et al.*, 2008), it is possible that the observed effects of low R : FR on MeJA-induced VOC emissions occur through modulation of JA signalling. However, how low R : FR conditions down-regulate the emission of constitutive VOCs remains elusive.

### Low R : FR light conditions affect MeJA-induced gene expression and VOC emissions

Most VOCs that are emitted upon MeJA treatment were also found after application of JA (Van Poecke &Dicke, 2002). Gene expression data for *BSMT1* and *TPS4*, which are involved in the biosynthesis of methyl salicylate and TMTT, respectively, showed a clear suppression of the MeJA-induced up-regulation when combined with low R : FR, which is consistent with the reduced emissions of MeJA-induced methyl salicylate and TMTT*. CYP72A13* is involved in converting geranyllinalool to TMTT (Bruce *et al.*, 2008), but the reduced MeJA-induced TMTT emission under low R : FR conditions was not accompanied by reduced *CYP72A13* transcript levels. It is possible that, under low R : FR conditions, the reduction in TMTT emission is caused by a reduction in the production of geranyllinalool, which is considered to be the rate-limiting step in TMTT emission (Herde *et al.*, 2008). Another MeJA-inducible gene involved in VOC biosynthesis that was not affected by low R : FR conditions was *TPS3* (Fig.6), which encodes a terpene synthase involved in the synthesis of β-ocimene and β-myrcene (Van Poecke *et al.*, 2001; Faldt *et al.*, 2003). Indeed, the MeJA-induced emission of trans-β-ocimene and β-myrcene also was not affected by low R : FR (Fig.5).

### Why are not all MeJA-induced VOCs suppressed by low R : FR?

The observation that the emission of not all MeJA-induced volatile compounds was reduced under low R : FR might be attributable to the occurrence of different signal transduction branches in the JA-mediated pathway and the role of ethylene in these branches. Upon herbivory or wounding, a signal transduction branch is activated that is under the control of the basic helix-loop-helix transcription factor MYC2 (originally known as *JIN1*;*JASMONATE INSENSITIVE1*), which negatively regulates a second branch of the JA pathway that involves the Ethylene Response Factor1 (ERF1) transcription factor (Verhage *et al.*, 2011; Pieterse *et al.*, 2012). The ERF1 branch of the JA pathway is mainly effective in inducing resistance against necrotrophic pathogens (Pre *et al.*, 2008). It is possible that the low R : FR-induced production of ethylene leads to enhanced induction of the ERF1 branch of the JA pathway, at the expense of the MYC2 branch. If the MYC2 branch were most effective in inducing VOC emission, this could subsequently explain the observed down-regulation of emissions of a number of MeJA-induced VOCs in low R : FR. However, it has been demonstrated that application of exogenous 1-aminocyclopropane-1-carboxylic acid (ACC; a precursor of ethylene) can have a positive effect on the emission of particular JA-induced VOCs in lima bean plants (*Phaseolus lunatus* L.) (Horiuchi *et al.*, 2001). Future studies could include genetic approaches, such as including *myc2* and *erf1* knockout mutants, but also the *phyB* (Phytochrome B) phytochrome mutant with disturbed R : FR signalling, in order to identify which signal transduction branches are involved in low R : FR-mediated control of MeJA-induced VOC emissions.

### Can VOCs serve as neighbour detection signals in dense stands?

The increase in ethylene emission makes this compound a suitable candidate signal that could mediate neighbour detection. Indeed, it has been shown that ethylene emissions are enhanced when *A. thaliana* plants grow at high density (De Wit *et al.*, 2012), that ethylene levels can accumulate inside dense tobacco stands to physiologically relevant levels, and that the perception of ethylene is required for tobacco to successfully compete for light (Pierik *et al.*, 2003, 2004). It is possible that this involvement of ethylene in plant–plant signalling at high densities relies on the up-regulation of emissions in response to neighbour-derived light signals, which is in sharp contrast to other VOCs whose emissions are reduced in response to the presence of neighbours and neighbour-derived changes in light quality. Nevertheless, it is still possible that VOCs are used to detect neighbours at a stage before the occurrence of light signals, which in *A. thaliana* stands is any developmental stage before the onset of touching of leaf tips between neighbouring plants (De Wit *et al.*, 2012). In *A. thaliana* canopies, there is only limited air volume within the shielded canopy where VOCs might be expected to accumulate. Thus, even though interplant distances in dense stands are very small, we propose that the reduced emissions make VOCs less likely to be reliable cues for detection of neighbours. Interestingly, the emission of a number of volatile compounds, such as (*E,E*)-α-farnesene, was not drastically down-regulated. This would lead to an altered composition of the volatile blend that is emitted under low R : FR conditions. It has to be investigated whether the altered composition of the volatile blend has an effect on proximate neighbours, as the absence of specific (groups of) VOCs can lead to a stronger response to other VOCs (Paschold *et al.*, 2006).

### VOC-mediated plant–herbivore interaction is affected by low R : FR light

In addition to signalling between neighbouring plants, VOCs also affect members of higher trophic levels (Dicke &Baldwin, 2010). As animal responses to VOCs are usually dependent upon the composition of the blend, rather than individual compounds (De Boer *et al.*, 2004; Van Wijk *et al.*, 2011), our observations can have consequences for interactions with animals such as herbivorous insects and their natural enemies. Possible effects of low R : FR conditions on the role of VOCs in attracting/repelling insects were studied here using the specialist herbivore *P. brassicae*. Although under control light conditions neonates of *P. brassicae* had a preference for MeJA-treated plants compared with control plants, this preference was lost when *P. brassicae* was allowed to choose between low R : FR-grown plants and low R : FR-grown plants treated with MeJA. Although preference for MeJA-treated plants seems to be counterintuitive, several examples of preference for volatiles of herbivore-infested plants have been reported previously (Bolter *et al.*, 1997; Kalberer *et al.*, 2001). The *P. brassicae* preference under control light conditions for MeJA-induced over mock-treated plants might be explained by the observation that MeJA-treated plants emitted higher amounts of volatiles and thus the signal was stronger and possibly more attractive. The finding that low R : FR-grown MeJA-treated plants were preferred over MeJA-treated plants implies, however, that the total amount of volatiles is not the only important factor. It is possible that, in this particular choice, the preference of neonate caterpillars is also affected by the composition of the blend, which is different between high and low R : FR-exposed MeJA-treated plants. In summary, caterpillar choice is probably affected by both the total amount of VOC emissions and the relative composition of the emitted VOC blends. Both can be affected by MeJA, low R : FR and their combination, and this probably affects herbivore preference.

### Conclusions

We found that shading had a negative effect on the amount of VOCs emitted by *A. thaliana*. Both constitutive volatile emission and MeJA-induced volatile emission were reduced by low R : FR conditions. This reduction in emission was mostly consistent with differences in gene expression in the biosynthetic pathways of different volatile compounds and seemed to have consequences for host selection by a specialist herbivore. As under natural and agricultural conditions plants most frequently grow in conditions with reduced R : FR ratios caused by proximate neighbours, it is possible that VOC-mediated multitrophic interactions in nature are different from those observed under growth chamber or glasshouse conditions focussing on individual plants. Both herbivores and predators or parasitoids of herbivores might be less capable of locating their hosts, because of the reduction in volatile emissions and changes in the composition of the volatile blend (Dutton *et al.*, 2000; Müller & Hilker, 2000) as a result of shading. We argue that these data emphasize the need for specific attention to light quality in VOC research on plant–plant and plant–insect interactions. The data presented here indicate that plant performance at high densities may be dominated by phytochrome signalling of light quality, thereby affecting plant growth, plant–plant signalling and plant–insect interactions.

## Supporting Information

Table S1 Primer sequences of primers used for RT-qPCR
