# Supplementary material for: Canopy light cues affect emission of constitutive and methyl jasmonate-induced volatile organic compounds in Arabidopsis thaliana
Source: New Phytol. 2013 Jul 12;200(3):861–74. doi: 10.1111/nph.12407 (PMC4283982; doi:10.1111/nph.12407)
Supplement: Table S1 — Primer sequences of primers used for RT-qPCR [file nph0200-0861-SD1.pdf]

Table S1: Primer sequences of primers used for RT-qPCR

**Gene**	**Primer**	**Sequence**
UBQ5	F	ACATCCAGAAGGAATCGACG
	R	CTTGATCTTCTTCGGCTTGG
TUB	F	ATAGCTCCCCGAGGTCTCTC
	R	TCCATCTCGTCCATTCCTTC
VSP2	F	ATGCCAAAGGACTTGCCCTA
	R	CGGGTCGGTCTTCTCTGTTC
BSMT1	F	TGGTCACTACTACGAAGAAGATG
	R	GAGCATTGGTTCACTAACAGC
TPS3	F	GCCACCATCCTCCGTCTC
	R	CCAAGCCACACCGATAATTCC
TPS4	F	TCGCAGCACACACCATTG
	R	GAGCAGCACGGAGTTCATC
